# Exploring the Possibility of Peak Individualism, Humanity's Existential Crisis, and an Emerging Age of Purpose

**DOI:** 10.3389/fpsyg.2017.01478

**Published:** 2017-09-05

**Authors:** Gabriel B. Grant

**Affiliations:** Yale School of Forestry and Environmental Studies, Yale University New Haven, CT, United States

**Keywords:** purpose, self-determination, cultural change, existential, meaning, millennials, adult development, culturomics

## Abstract

There is an emerging cultural narrative in the United States that we are entering an age of purpose—that millennials, more than any other generation, are searching for purpose and purposeful work (Sheahan, 2005) and that we are entering an era or economy of purpose (Hurst, 2014). For profit, non-profit, and educational institutions are perceiving and adapting to serve millennials' demand for purpose in life, specifically within the workplace (Klein et al., 2015). Yet, longitudinal studies of purpose do not exist, and millennials are also referred to as GenMe. Existing quantitative research suggests they (we) are increasingly individualistic, materialistic, and narcissistic (Greenfield, 2013). Google's digitization of millions of books and the Ngram Viewer allow for quantified analysis of culture over the past two centuries. This tool was used to quantitatively test the popular notion that there is a rise in demand for purpose. Analysis reveals a growing interest in purpose-in-life and a shift toward collectivistic values emerging over the lifespan of the millennial generation.

## Introduction

Purpose is defined as “a stable and generalized intention to accomplish something that is at the same time meaningful to the self and consequential [beneficial] for the world beyond the self” (Damon, 2008). Over the past decade, we have learned a great deal about the correlative benefits of purpose. A sense of purpose may extend one's life (Hill and Turiano, 2014), and, simply put, make life worth living. Psychological and physical wellbeing, resilience, ability to cope, motivation, life satisfaction, positive affect, reduced stress, reduced rates of Alzheimer's disease and cognitive impairment, reduced inflammation response, improved cardiovascular and metabolic markers, are all benefits of having a sense of purpose (Bronk, 2013).

And, there is much we do not yet know. Purpose measures do not include a beyond-the-self component (Crumbaugh and Maholick, 1964; Hill et al., 2016), so we do not know what benefits are associated with a beyond-the-self or prosocial purpose (e.g., I want to heal people) vs. an individual purpose or life goal (e.g., I want to drive race cars). There is a general lack of longitudinal research. As such, the causality of benefits is undetermined. Are people healthy because they have purpose or do they pursue purpose because they're healthy? We are just beginning to explore and gather evidence-based practices for fostering or supporting purpose. While there are many theoretically grounded recommendations for cultivating purpose, empirically evaluated purpose interventions are few and far between. Nevertheless, there is an academic field emerging and correlative benefits identified within available research supports the age-old wisdom that purpose is worth striving for and worthy of study (Bronk et al., 2009; Hill et al., 2016).

Purpose also appears to be a trending focus of organizations. In 2016, the Academy of Management themed their 76th annual meeting “Making Organizations Meaningful.” The big four accounting firms, PwC (formerly PricewaterhouseCoopers), KPMG, Deloitte, and EY (formerly Ernst and Young) are all investing in purpose development in their workforce (Klein et al., 2015). EY, for example, created the Beacon Institute, a think tank focused on transforming business through the science of purpose; hosted a roundtable meeting on purpose at the 2016 World Economic Forum, and sponsored a Harvard Business Review study of how executives are integrating purpose throughout their organizations (Harvard Business Review Analytic Services., 2015). LinkedIn and Imperative also conducted a study on the role of purpose in the workplace (Hurst et al., 2016). PwC and the CEO Force for Good have launched a multi-year initiative to examine how purpose can contribute to employee performance, tenure, and fulfillment (CECP., 2017). And, a recent study surveyed MBA students regarding their preference for or willingness to take lower salaries to work at companies that most benefitted the world (Franceschini et al., 2015). Similar institutional trends toward serving the perceived demand for purpose can be found within non-profit and academic institutions (Klein et al., 2015). These organizational initiatives and research studies have been launched in response to what the organizers or authors perceive as an emerging increase in demand for purpose in life even though longitudinal data is not yet available in support of such perceptions.

By contrast, existing empirical research suggests the millennial generation is the most individualistic (Greenfield, 2013), narcissistic (Twenge et al., 2008), and the least altruistically motivated (Twenge et al., 2010). Moreover, they/we are suffering from the correlative psychopathologies, e.g., depression (Twenge and Foster, 2010). How is it then that in the midst of the most individualistic culture measured (Greenfield, 2013), we have a competing narrative that we are entering an age of purpose? How can the millennial generation be colloquially known both as GenMe and GenWe? Are emerging university and corporate interests in purpose a marketing fad we may expect to come and go? Or are they part of a larger cultural shift? How would we know?

## Methods

This study uses Google Books Ngram Viewer to test the hypothesis that interest in or demand for purpose in life is on the rise. By indexing frequencies of words and phrases over the past 200 years, we are able to explore and test for cultural change that would be indicative of entering an age, era, or economy of purpose. Our existing age, the information age, facilitates the collection of big data including our ongoing communications and the digitization of recorded history (e.g., tweets, emails, blogs, books, articles, academic publications). Measuring purpose in one's life is a new endeavor and, while there are no longitudinal studies of purpose that would allow us to compare the presence of purpose from generation to generation, we can investigate cultural trends by quantitatively analyzing digitized texts. This analysis of big data to study human culture is a research method coined culturomics (Michel et al., 2011) and has been used to explore the changing psychology of culture leading up to the new millennium (Greenfield, 2013).

Over the course of written history, data grows more robust because more is published and more of what is written is digitized and available to analyse. Moreover, as literacy increases and publishing grows cheaper and more prevalent, we may expect the available data to be increasingly democratized or representative of a whole population. As we take our conversations online and public, we create even more democratized data sources that capture more and more voices. For example, today scientists have demonstrated analysis of twitter tweets to measure happiness (Dodds et al., 2011). This type of analysis could provide real time measures of happiness and/or other values of interest (e.g., purpose) within communities or populations. Researchers are exploring that now, but given Twitter and other services are relatively new, we can't yet explore historical cultural trends using those data sources. What we do have available is Google's digitized book corpus or Ngrams which indexes words and phrases from books published as early as the year 1,500.

### Ngram sample

Google's Ngram viewer allows for the quick analysis of cultural trends within a massive corpus containing 5,195,769 digitized books or about 4% of all books ever published. The books were collected through Google's digitization of over 40 university library collections that began in 2004 and through 30,000 partner publishers who directly submit books for digitization (Michel et al., 2011). The Ngram viewer displays the frequency of words or short phrases (up to five words long) as they are used over time. Where there is ample data we can view smooth robust cultural trends of frequently used words and phrases. By contrast, infrequent words or phrases and small sample data generate more erratic results. With a smaller sample of books, each occurrence of a word or phrase more heavily impacts the result. Publishing was rare in the 16th and 17th centuries, so analyzing the data before 1,650 tends to generate highly erratic results with series of spikes rather than clear trends. Analyzing the data from 1,650 to 1,750 provides somewhat erratic results. However, data after 1,800 is sufficient to clearly view trends of moderately popular phrases such as “purpose in life.”

For this research, analyses includes years 1800-2008, because there is sufficient data to show the context leading up to and the culture during the millennial generation. Millennial is used commonly to refer to anyone born in the 1980's or 1990's, but can sometimes include people born in the early 2000's. This analysis ends at 2008 because today the Google Ngram corpora only include data through 2008.

Given the Google books collection was first created in 2004 both by digitizing library collections and by inviting publishers to directly submit manuscripts, older books in the corpora will be more likely than new books to come from library collections and newer books are more likely than older books to be collected through publisher partnerships. Thus, in addition to reflecting cultural changes in word frequency use, results may be influenced by library selection process biases. Moreover, the force of this possible library selection bias diminishes over time as libraries are removed as an intermediary and publishers add books directly to the corpora. To help control for this shift in sampling and resulting impacts on the corpora composition, an “English 1M” corpora was created containing only one million books that have been re-sampled to exhibit a representative subject distribution of all published books as reflected by their BISAC subject codes. For this research, analyses are conducted using the full corpora and verified when applicable using the English 1M corpora to help detect whether observed trends may be a sampling effect from how the corpora was constructed.

Finally, the Google Ngram corpus includes several corpora of various languages (e.g., Chinese, Spanish, German, and French), and separate corpora for English books published in the USA and English books published in the UK. This allows us to begin using an English cultural analysis and then to explore for similar trends across varying languages and geographies.

### Analysis of historical trends in phrase frequency

Ngram viewer plots the frequency of words over time by dividing the number of instances of a word by the total number of words in that year. To calculate the popularity of a phrase, the Ngram viewer divides the number of instances of the specific n-word phrase by the total number of instances of n-word phrases (e.g., a 3-word phrase would be normalized by dividing the instances of the specific phrase that year by the total number of instances of all 3-word phrases in that particular year).

It is necessary to select popular words or phrases that are culturally relevant for our research purposes because words or phrases with less than 40 instances in a given year are omitted from the corpus to prevent the dataset from growing unmanageably large.

For our analysis to be the most meaningful, words or phrases must have a relatively narrow range of interpretations such that their use in the data closely represents the intended underlying concept and the frequency of alternate or misrepresentative uses is relatively low. Words or phrases with many meanings will return results that are unrelated to the intended value or concept under investigation. Ngram viewer provides an easy tool to search within Google Books to see the use of words or phrases in context such that one can evaluate whether the actual use of the words or phrases in the data is aligned with the underlying research idea. Further, by analysing synonymous words or phrases when available, one can see whether there is a similar pattern present and thus whether there is a cultural trend in the underlying concept or idea that transcends any particular word or phrase.

## Study overview

This study applies culturomics using the best available data to explore five research questions. (1) What words or phrases, as they are used in popular literature, best represent the concept of purpose? And, using culturomics and the Google Ngrams corpora to analyse the frequency of those words or phrases, how has interest in purpose changed over the past two centuries? (2) Are patterns of cultural interest in purpose within popular literature replicable within scholarly literature? (3) How does interest in purpose differ among cultures around the globe? (4) How do patterns in the cultural popularity of purpose relate to patterns in cultural expressions of individualism and collectivism? (5) How do patterns in the cultural popularity of purpose, individualism and collectivism relate to cultural popularity of religion or spirituality?

## Analyses and findings

### There is an unprecedented growth in popularity of “purpose in life” throughout the lives of the millennial generation

Searching for “purpose in life” in Google books shows that this particular phrase reflects the target concept of a purpose as described by Damon et al. (2003) and Damon (2008) and includes a “beyond the self” or “prosocial” component that differentiates the concept from an individual life goal. Google books allows quick sampling of terms and phrases to assess their meaning as they are used in context of the books that make up the Ngrams corpora. For example, out of 100 instances sampled to represent a distribution of use over time, only twice was the phrase “purpose in life” used to mean something that was clearly different from the sought after meaning. Both of those instances were biology related and used “purpose in life” to mean mating or reproduction. Eighty-three instances clearly reflected the intended cultural idea of purpose. Fourteen instances lacked sufficient explanation within the available snippets or book descriptions to differentiate whether the phrase was used to represent a prosocial purpose beyond an individual goal.

Compared to the phrase “higher purpose,” “purpose in life” was more likely to consistently refer to an individual's “purpose in life.” In the ninteenth century, “higher purpose” often refers to the higher purpose of a thing and sometimes, more recently in the twenty-first century, “higher purpose” is used in reference to an organization's purpose. Generally, “higher purpose” becomes a strong indicator of the intended concept as it is generally used during the twentieth and twenty-first century, once references to the higher purpose of things subside. The phrases “life purpose” and “life's purpose” were also found to be close synonyms for “purpose in life.” The phrase “purpose driven,” for example, was found to be less appropriate because of the wide variety of uses or meanings. The word “purpose” on its own would include all of the phrases above, however it has the widest variety of meanings and uses and thus was found to be a less than appropriate indicator for measuring the concept of interest.

**Figure 1:**
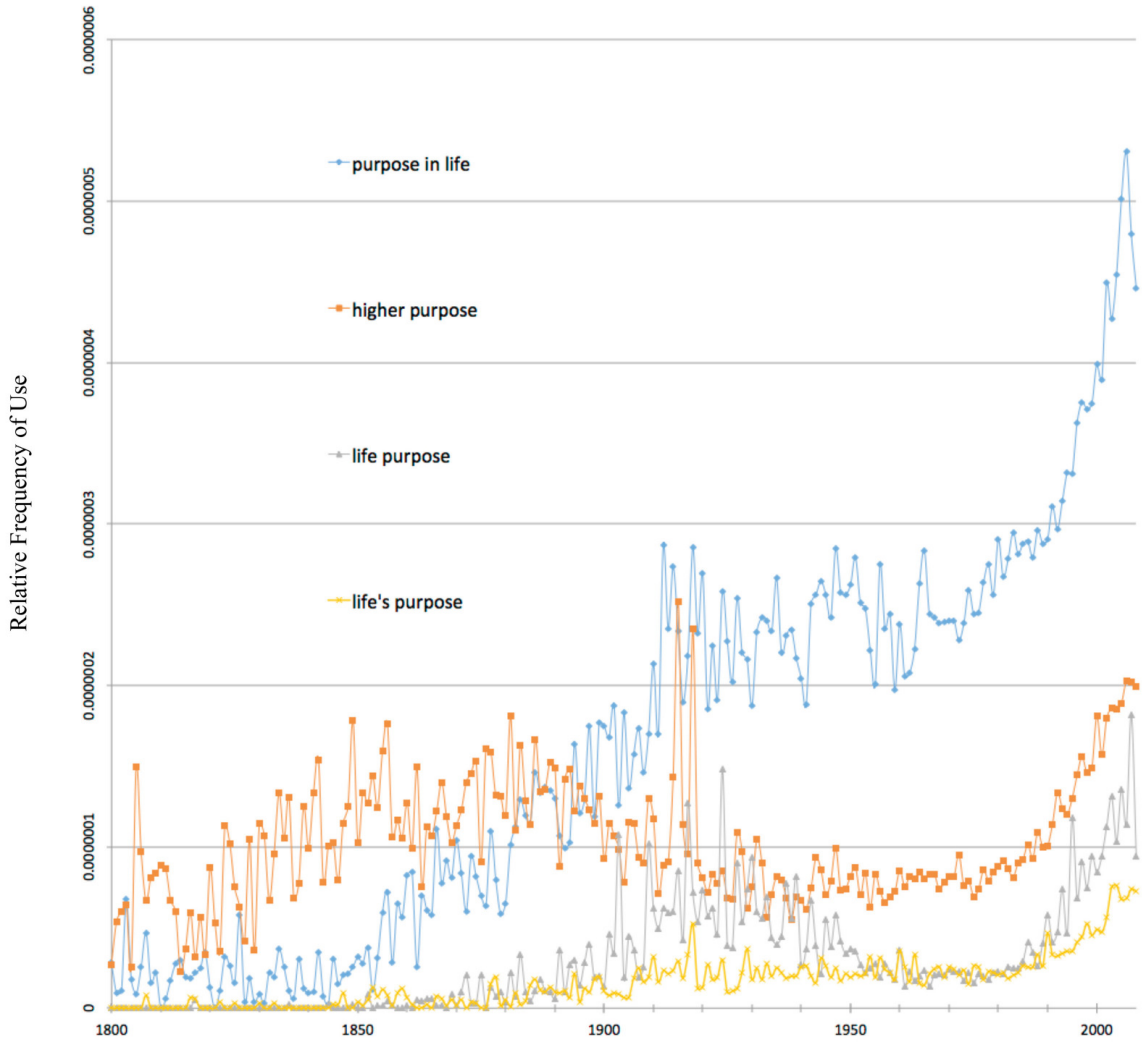
Frequency of the phrase “purpose in life” and synonymous phrases in the Google corpus of English books from years 1800-2008.

As seen in Figure 1, there is increasing popularity of the phrase “purpose in life” from 1850 until approximately 1915 at which point popularity plateaus. It begins to climb again just before and throughout the 1980's. Then, around 1990 the trend accelerates and sustains an unprecedented climb that continues to its peak popularity in 2006. Phrases synonymous to the phrase “purpose in life,” including: “higher purpose,” “life purpose,” and “life's purpose,” produce similar cultural Ngram trends, peaking in the early 1900's and then climbing to their peak popularity at around the end of our available data. “Life's purpose” and “life purpose” come into use in the twentieth century and thus are relatively newer phrases compared to “purpose in life.” Given the synonymous phrases' patterns of relative frequency replicate the original phrase, and that these results are consistent across both the English corpora and the English 1M corpora, this finding is considered to represent the cultural popularity of the intended underlying concept of purpose.

### In addition to the popular literature, interest in “purpose in life” is also trending within scholarly literature

**Figure 2:**
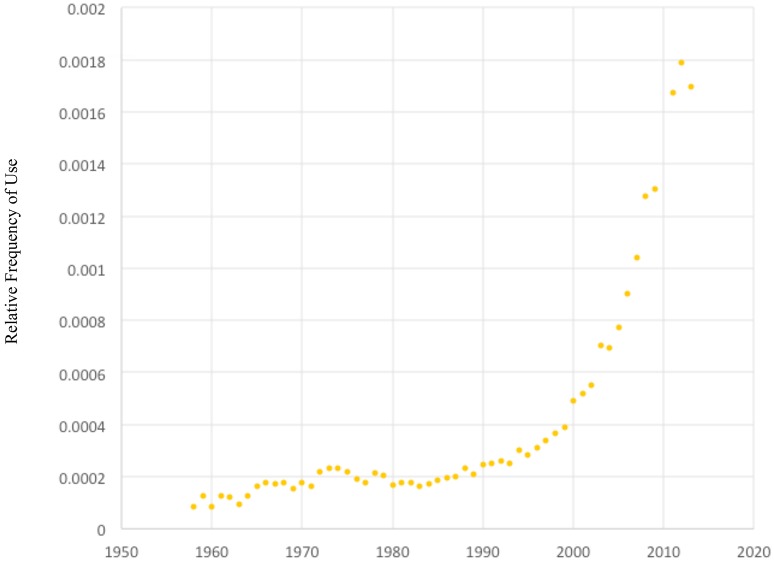
Frequency of academic publications containing the phrase “purpose in life” in a Google Scholar search from years 1960-2013.

A second analysis was designed to test whether the trending interest in“purpose in life” was replicable in academic literature or limited to popular literature. Using Google Scholar, we can measure the number of academic publications that use the phrase “purpose in life” within each year of publication. Dividing by the total number of publications available in that specific year gives a frequency of publications containing our specific phrase analogous to the Ngram analyses of popular literature. Figure 2 shows exponential growth in academic interest in “purpose in life” during the first decade of the new millennium. While the number of publications containing the phrase “purpose in life” rose from 6150 in 2012 to 6340 in 2013, the total number of publications in the database rose faster, such that our graph of the frequency shows a decline from year 2012 to 2013. This growth starts near 1980 and then accelerates around 2000, or approximately the time the first millennials would have begun attending college.

### The unprecedented growth in the popularity of “purpose in life” is strongest in us english, but “purpose in life” is also trending upwards in french, spanish, and british english over the lifetime of the millennial generation

**Figure 3:**
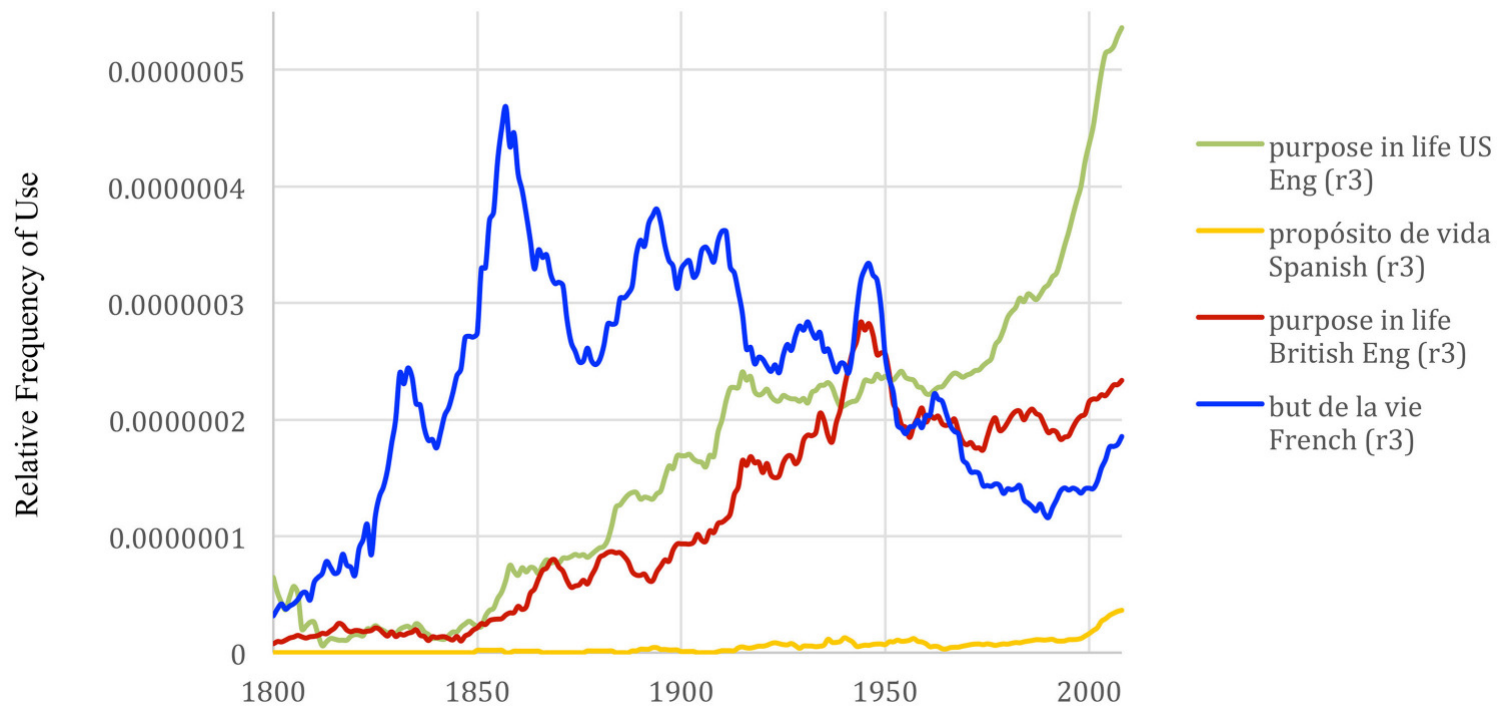
Frequency of the phrase “purpose in life” in French, Spanish, US and British English in books from years 1800-2008. The graph was made with the Google Books Ngram Viewer with a smoothing of 3.

Google Ngrams allows the analysis corpora of several languages, such that we can investigate whether “purpose in life” is trending in other cultures. As can be seen in Figure 3, popularity of “purpose in life” is increasing between 1980 and 2008 in US English, British English, French, and Spanish, however the increases are not nearly as significant in other places as can be observed in the US. The concept is notably oldest in the French language and relatively young in the Spanish language. The concept of purpose in life does not translate directly into German. The “meaning of life” is the closest related concept, but “meaning of life” is distinct in the English, French, and Spanish languages and is worthy of a study unto its own.

**Figure 4:**
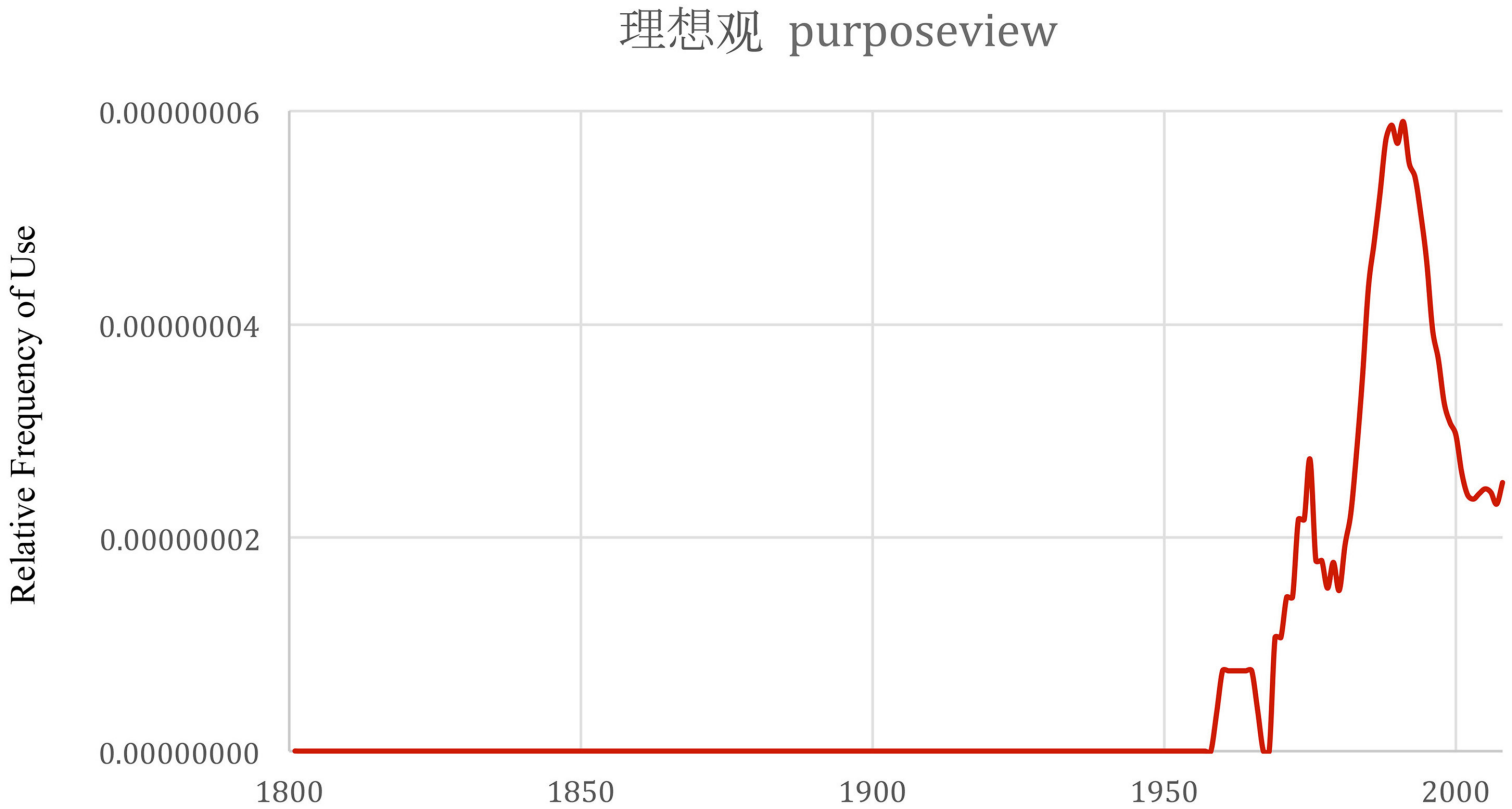
Frequency of the phrase 理想观 or purposeview in Chinese books from years 1800-2008. The graph was made with the Google Books Ngram Viewer with a smoothing of 3.

Chinese is quite distinct. The relationship to purpose, rather than being emergent or coming from within, is often experienced as a top down commandment, a dictated responsibility to assimilate one's aim or role in contributing to society. In 1980, Deng Xiaoping proposed Chinese education be organized around four must-have character traits - Chinese citizens in the new era were to be purposeful, moral, literate, and disciplined. As a result of this directive, several purpose related concepts come into existence in the Chinese corpus or grow very quickly in the year 1980, and then exhibit a sharp decay shortly thereafter. Nevertheless, one concept that emerged during that time, 理想观, translates to purposeview, shown in Figure 4. Like worldview, the concept of purposeview opens an entire domain of how one thinks about or experiences purpose. In our short exploration of how different cultures express and experience purpose in life, we were delighted to uncover a word and concept to represent this domain of inquiry.

### We may have the most individualistic, materialistic, narcissistic culture that has ever existed. however, trends toward individualism may have plateaued or peaked. most recent data illustrates a swing back toward collectivistic values

In “The Changing Psychology of Culture from 1800 through 2000” Greenfield (2013) uses Google Ngrams to quantitatively test and demonstrate her theory of ecological change as a driver of values, behaviors, and psychology which predicts that with “urbanization, increased wealth, technological development, or greater availability of formal education—values, behaviors, and psychology become more individualistic and materialistic” (Greenfield, 2013). As we move away from the village, our survival is dependent more on our individual performance, individual accumulation of possessions, etc., than it is dependent on a particular community. Greenfield plots words like “choose” and “decision,” which are associated with individualistic values and meaning alongside words more associated with collectivistic values and meaning like “obliged” and “duty” As expected, the individualistic word popularity in the English language significantly trends upwards during the 19th and 20th centuries. Meanwhile, the words associated with collectivistic values trend downward.

Greenfield's analysis suggests our U.S. and global cultures are perhaps the most individualistic they have ever been. However, this investigation is specifically interested in what is happening during the millennial generation. Rerunning Greenfields experiments to include data through 2008 produces a notably different finding^1^.

**Figure 5:**
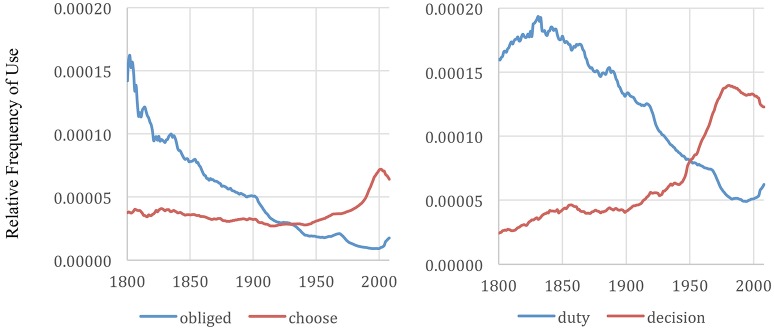
Frequency of “obliged” and “choose” and “duty” and “decision” in English books expanded to include data from 2000-2008. The graphs were made with the Google Books Ngram Viewer with a smoothing of 3.

**Figure 6:**
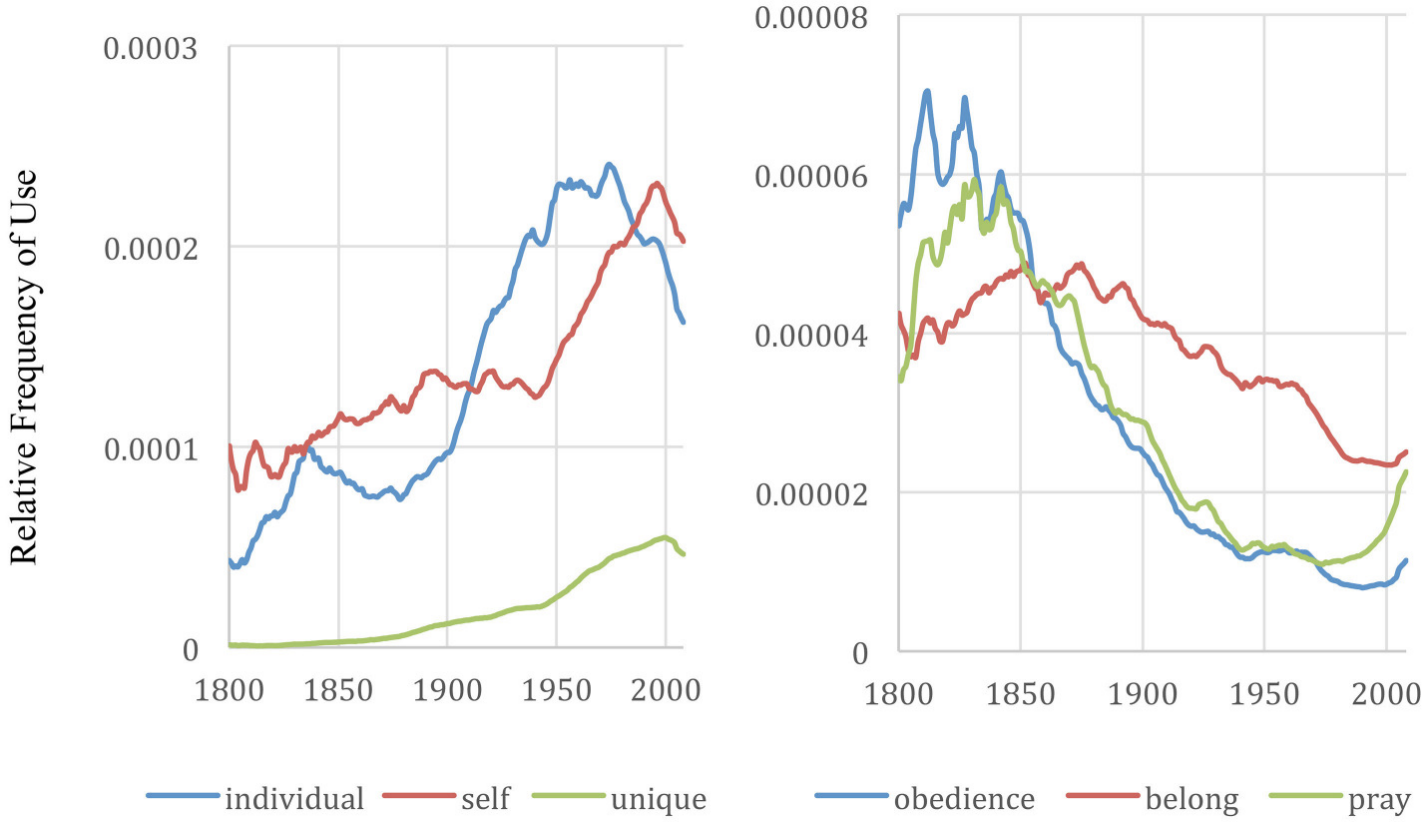
Frequency of “individual,” “self,” and “unique,” and “obedience,” “belong,” and “pray” in English books expanded to include data from 2000-2008. The graphs were made with the Google Books Ngram Viewer with a smoothing of 3.

Using Greenfield's (2013) culturomic indicators of individualistic/collectivistic culture, within the English literature, there appears to be a peak individualistic period and a trend reversal back toward collectivistic values within the lifetime of the millennial generation. This inflection point or trend reversal is visible in the majority of Greenfield's (2013) indicators. Twenty-five of her 28 chosen words show a clear trend toward individualism throughout the nineteenth and twentieth century. Twenty of the 28 words are trending away from individualism toward collectivism since their 1980 values. See examples in Figures 5, 6. These results are consistent across both the English corpora and the English 1M corpora. The frequency over time of all 28 indicators is shown in the Appendix (Supplementary Material; using a logarithmic scale such that it can be done in as few charts as possible).

**Figure 7:**
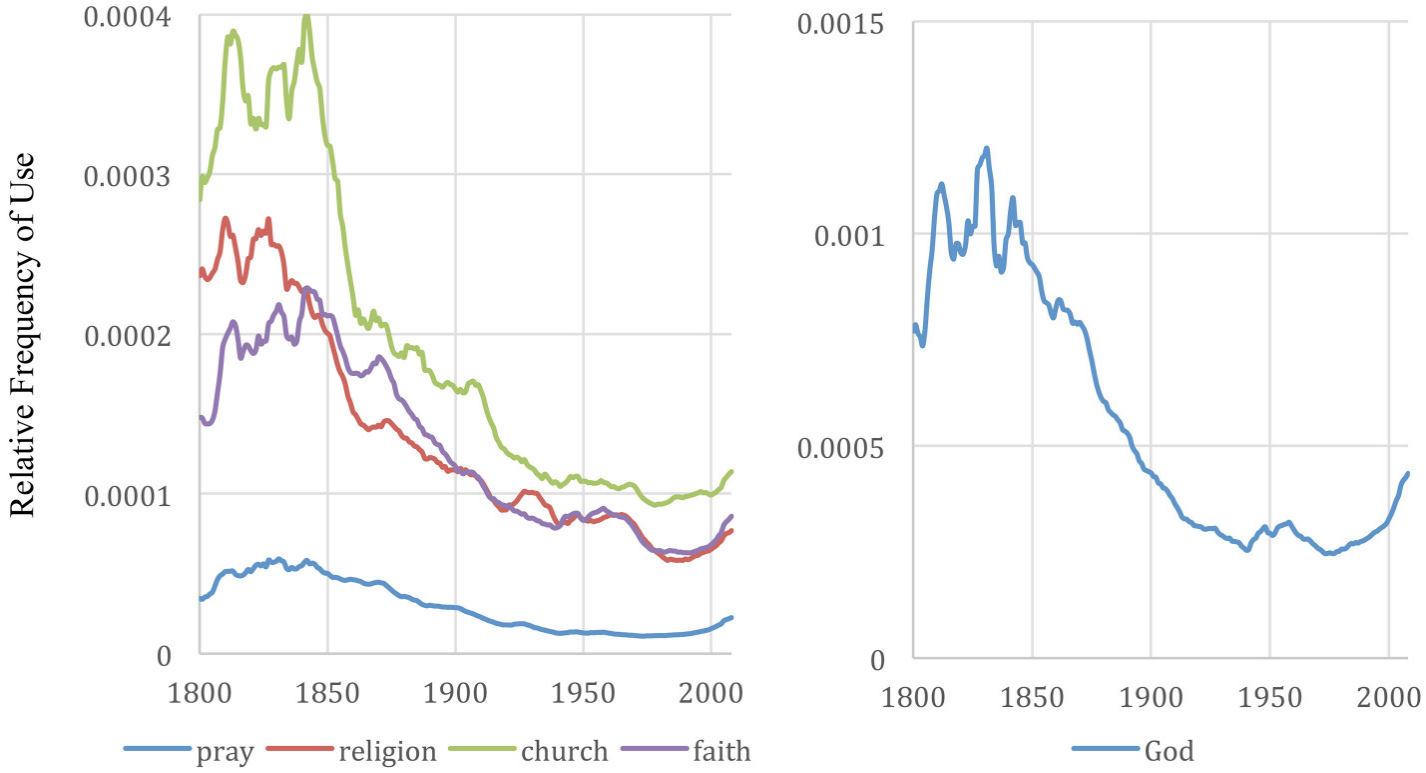
Frequency of “pray,” “religion,” “church,” “faith,” and “God” in English books from years 1800-2008. The graph was made with the Google Books Ngram Viewer with a smoothing of 3. God is displayed using a different scale because, while the trend over time is similar, the word's overall use is far more frequent than the other indicators.

### Popularity of spiritually related concepts reached an all-time low around 1980 and has since been trending upward

Spirituality and religion are known pathways for developing purpose (Tirri and Quinn, 2010), a frequent theme among people's conceptions of purpose (Hill et al., 2010; Moran, 2014), and are a frequent theme among books identified in Google books searches for “purpose in life” and synonymous phrases.

Existential thinkers distinguish four basic dimensions of human existence—physical, social, psychological, and spiritual. Greenfield's theory focuses on how changes in the physical dimension correspond with changes in the other dimensions. Greenfield's (2013) attention to individualistic and collectivistic values, authority, belonging, duty, conformity and power relate to the social dimension. The attention to choice, actions, feelings, ego, and self-relate to the psychological dimension. The spiritual dimension is least explored in her study, however the mention of “pray” and the significant jump during the millennial generation (seen in Figure 6) piqued my interest.

Exploring spiritually related terms using Google Ngrams results in a clear trend using a variety of indicators, e.g., God, faith, spirituality, prayer, religion, worship, church, etc. The popularity of spiritual terms falls over the nineteenth and twentieth century reaching their lowest popularity in about 1980 at which point, they level off and begin to grow until 2008 when our data ends, analogous to the trends exhibited by the psychological and social indicators.

## Discussion

The results of this study are summarized in five key findings. First, there is an unprecedented growth in cultural popularity or interest in “purpose in life” throughout the lives of the millennial generation. Second, beyond being a growing topic of interest in popular literature, “purpose in life” is also a fast growing topic within scholarly research. While purpose is an age-old phenomenon of inquiry, modern interest in American English culture is at a historical peak. Third, there is some evidence to suggest that the popularity of “purpose in life” is strongest in American English culture, but also trending upwards in other cultures as indicated by data from French, Spanish, and British English corpora. Fourth, looking back over the past two centuries of written American English history gives us the impression that we are at the height of individualism, thus millenials may be appropriately called Gen-Me. However, looking back over a shorter time horizon, such as the past 30–40 years, may leave us with the experience that, we are witnessing Gen-We, individualism plateaued or peaked and millenials are coming to age as part of a culture that's trending toward collectivistic values. Fifth, the 1980's appear to be a time of peak individualism marked by social and psychological indicators and religious or spiritual indicators. As such, this time period may represent humanity's existential crisis. Furthermore, religious and spiritual indicators have similarly been on the rise since alongside social and psychological collectivistic indicators over the recent years given available data.

Greenfield's (2009) theory linking ecological change with changes in human development demonstrates how value systems, behaviors and psychologies adapt to fit changes in physical environment or ecology. Whereas, collectivistic values of the pre-industrial society are driven by the need to succeed as a village or tribe, those collectivistic values give way to individualistic values as industrialization and urbanization come into being and one's success increasingly relies on individual performance. Provided we continue to urbanize, increase wealth, develop technologically, and participate in formal education, Greenfield's theory predicts that we may expect this trend to continue and indeed, that is the pattern observed using cultoronomics indicators during the nineteenth century and nearly to the end of the twentieth century (Greenfield, 2013).

The recent data explored in this study, however, illustrates a societal values inflection point and acceleration in the opposite direction. Trends toward individualistic values are leveling off or reversing between the years 1980 and 2000. And, an unprecedented interest in purpose is emerging over the same time period. This begs the question: Are there changes in ecologies or physical realities that would inspire a shift back toward collectivistic values and a new search for purpose?

Constructive developmental theories suggest there are. Many different people explore various nuances of human development through different constructive developmental frameworks (Kohlberg, 1969; Kegan, 1980; Torbert, 1991; Cook-greuter, 2000), yet these theories parallel one another (Wilber, 2000; McCauley et al., 2006). There exists some agreement that three orders of development describe the meaning making systems of most adults (McCauley et al., 2006). Within the framework provided by McCauley et al., the first common adult stage is called the dependent order where individuals derive their sense of self from their relationship to others. This stage is associated with a socialized mind and correlates with motivation to conform and a sense of duty. The next stage is called the independent order, where humans begin to reflect on the collective and in so doing, discover their independent identity. The third, inter-independent order, transcends and includes the independent and dependent orders. Humans begin to reflect on both their own selves and on the system. They can see themselves as both independent from and part of the larger whole. Individualistic values and collectivistic values can be held concurrently rather than at odds with one another. The dichotomy relaxes.

Similar to Greenfield's (2009) theory linking ecological change to human development, constructive developmentalists suggest our cognitive complexity or consciousness evolves to respond to the ecological challenges faced by our species (Kegan, 1995). The first two stages of consciousness in constructive developmentalist models are aligned with the two stages of development in Greenfield's (2009) theory of social change and human development. Human adaptation to rural environments prioritizes collectivistic values. Human flourishing is contingent on the collectivistic success of the village or tribe. With industrialization and urbanization, humans adapt to prioritize individualistic values and their flourishing is contingent on their individualistic success.

Constructive developmentalists differ from Greenfield by offering a third stage of human development (also referred to as consciousness or cognitive complexity) that is viewed as an adaptive response to challenges our species faces that are beyond the limits of or caused by the second stage of development (Kegan, 1995; McCauley et al., 2006). Such challenges include new levels of complexity (e.g., wicked problems) and problems created by our independent level of consciousness (e.g., the cold war, global warming). These problems require us to interact with complex systems and recognize interconnectedness while simultaneously reflecting on oneself as both autonomous and as part of the system. Moreover, while confronting one's own mortality can inspire an individual existential crisis, these problems introduce the possibility of confronting our mortality as a species.

Moving from one stage or order to the next involves reconstructing how we make meaning or understand the world. Our pursuit of purpose in life is a search for meaning and is a healthy human response during an existential crisis or collapse of meaning (Frankl, 2014). It is possible that our peak individualistic culture between 1980 and 2000 has triggered such a crisis on a societal scale, and inside that journey people are searching for purpose, as Frankl would have predicted and prescribed.

The concept of purpose in life may show up for people in various ways during various stages of their development. Purpose could simply be given to you by the role in society you're born into, purpose could be something you achieve or find, and it could be yours to create or self-author, or even to share in creating with others. The idea can include both a collectivistic and individualistic quality, both an expression of the autonomous self and a commitment to the greater world. Thus, while purpose appears to correlate with a third stage of development, people can interpret purpose differently in various developmental stages. This flexibility may make purpose a robustly useful tool for helping people lean into a healthy growing edge at any stage of development.

## Limitations and future research

There are several considerations to take into account when interpreting these results. First, it is important to keep in mind that books published between 1980 and 2008 are likely authored by members of older generations, not millennials themselves. Thus, while there is a clearly increasing cultural trend around the idea of purpose in life and a turn toward collectivistic values during the living years of the millennials, one could argue that the data presented here is more likely a reflection of changes in people who are in a later life stage than millennials themselves. This culture is likely to influence millennials, but is not necessarily the product of or a direct reflection of the millennials (they/we still could be GenME).

An alternative view could recognize that millennials both exist within and co-create the culture such that distinguishing these cultural trends as pre-millennial is overly simplistic. For example, millenials are cooperating with their advisors, co-creating research agendas and contributing to the research on purpose in life that is exponentially growing as millenials enter into graduate school. And, we are being raised by a generation that is reintegrating collectivistic values and actively supporting the next generation (through literature and organizational initiatives) in pursuing purpose. Should millenials continue the trend, they may be well equipped to take it to the next level.

Second, what is published in books is not democratic or a random sample of the population. Authors may tend to be more educated, affluent, and have the luxury of allocating more time for thought and reflection than the general population. They may have an environment that was exceptional in supporting them to achieve higher than average levels of human development. They may be early adopters of cultural trends and ideas, perhaps early adopters of existential crises, or a search for purpose. They may over represent an affluent, well to do, educated class. Alternatively, the idea that pursuing purpose is an elitist idea may itself be an elitist idea. Researchers are often surprised to find people experiencing high levels of purpose and meaning in what many may consider to be “dirty” jobs (Wrzesniewski, 2014).

Third, Google Ngrams and our analysis of the academic literature treats all publications as being equal. Thus, we are not differentiating, for instance, between how many copies of any book were published, or what actually was read much less acted upon. This analysis would not tell us, for example, if books mentioning “success in life” were selling 100 times as many copies as those mentioning “purpose in life.” Each published book is counted equally in this method, regardless of actual sales or how often it was read. Google Ngrams has enabled a quick quantitative proxy for culture through our language, but this method has its limits and, for instance, falls short of exploring corresponding changes in our behavior.

Fourth, using single word indicators to explore dichotomies is as slippery as dichotomies themselves. A single word's meaning changes so easily given the context. In many instances “individualistic” words are used in collectivistic ways and vice versa. Exploring trends in word popularity from this level and adding interpretation to those trends should be met with healthy skepticism. Phrases allow us to target more accurately a specific concept. There are similar challenges, but far fewer of them.

Fifth, the academic literature distinguishes between searching for meaning and the presence of meaning in one's life or similarly, searching for purpose and identified purpose. The growing popularity of purpose in the academic and popular literature most likely indicates interest in or a search for purpose. The presence of a search for purpose could indicate a lack of purpose and/or contribute to the presence of purpose. These findings at best demonstrate a searching for purpose at a cultural or societal level, but they do not tell us whether or not people are experiencing purpose. Whether an experience of purpose in life is found or created and how our behavior is impacted remains to be explored.

Sixth, reframing the collectivistic to individualistic shift in a developmental framework creates a new context for interpreting research on the psychological or motivational attributes of a generation. For instance, a constructive-developmental theory framework lends hypotheses toward understanding what might cause us to evolve toward reintegrating collectivistic values. However, a much higher level of analysis would be required to determine whether the data we are seeing is an expression of the higher levels of consciousness. Research is not yet available for understanding expressions of purpose through a constructivist developmental lens. Nor does there appear to be any research yet that applies a constructivist developmental lens to historic texts to study our collective development over time.

Seventh, in Kegan (1995) research observations, the shift from the first (dependent) order toward the second (independent) order occurs in middle to late years of adulthood. Thus, current psychological studies that tend to sample high school and college age students may be less valuable in documenting and understanding these types of societal shifts that tend to be expressed during adult development.

Finally, taking the perspective of an expanded consciousness that can transcend individualistic and collectivistic values, this exploration was framed within a dichotomy of individual and collective. Purpose in life, to many, includes both a “self” and a “beyond-the-self” component. Care for the individual and the community are not mutually exclusive, but rather can be seen as being bound up in one another. Thus, a Gen-We or purpose based generation may not experience a dichotomy between individualistic and collectivistic, self and other. In other words, if I have integrated purpose into my identity than Gen-Me could also be Gen-We. Pursuing purpose, working toward solutions for the whole becomes my own self-interest. Exploring this possibility would require two-dimensional models that allow for the expression of ambivalence, or integration of individualistic and collectivistic objectives concurrently. Our world or purposeview may evolve to transcend assuming their juxtaposition in a one-dimensional space.

## Conclusions

Perhaps we are both. We are both the most individualistic, materialistic, narcissistic culture on record and we are historically the most interested in purpose in life. These results suggest that we could also become the first generation to reverse or transcend historical trends toward individualism. These results also support the possibility of an emerging purpose generation, era, or age. Should this trend continue, we may expect our institutions (e.g., corporations and universities) to further adapt and market themselves toward fulfilling a demand for purpose.

Our individualistic, materialistic, and narcissistic culture is oddly shaping out to be our greatest opportunity to pursue purpose. Purpose in life is emerging at the time you would least and most expect. In recognition of this macro level paradox I invite people to hold a similarly powerful context at the micro scale—to consider that each individualistic or materialistic thought of your own, or projected judgment onto someone else, is an opportunity to invite yourself and someone else into something a little more appealing, something a little more an expression of you, and a little more in service of someone else. Consider each experience of individualism an opportunity to explore something a little more purposeful. The time is right.

## Author contributions

GG is fully responsible for the authorship of this article. Several people inspired the work and assisted him with translation and they are acknowledged in the acknowledgment section of the article.

### Conflict of interest statement

The author declares that the research was conducted in the absence of any commercial or financial relationships that could be construed as a potential conflict of interest.

## Footnotes

^1^Trends in Figures 1,5–7 are displaying data from all books published in English, which is not noticeably different to displaying the data from books published in US English since US English books dominate the sample.
