# Supplementary material for: Exploring the Possibility of Peak Individualism, Humanity's Existential Crisis, and an Emerging Age of Purpose
Source: Front Psychol. 2017 Sep 5;8:1478. doi: 10.3389/fpsyg.2017.01478 (PMC5591862; doi:10.3389/fpsyg.2017.01478)

Exploring the Possibility of Peak Individualism, Humanity's Existential Crisis, and an Emerging Age of Purpose

Gabriel B. Grant

Yale University, New Haven, CT, USA

195 Prospect St, New Haven, CT 06511

gabriel@byronfellowship.org

Appendix

This appendix includes figures 1, 2, 3, and 4, that show the frequency of 28 individualistic and collectivistic indicators in English books from 2000-2008. The graphs were made with data from Google Books Ngram Viewer's English 1M corpora with no rounding and are plotted using a logarithmic scale to display in four charts 28 results that range widely in their Relative Frequency of Use. Explanation for how these indicators were chosen is available within of Greenfield's (2013) article, *The Changing Psychology of Culture From 1800 Through 2000*.

Figure 1, Collectivistic indicators that trend toward collectivistic values between 1980 and 2008

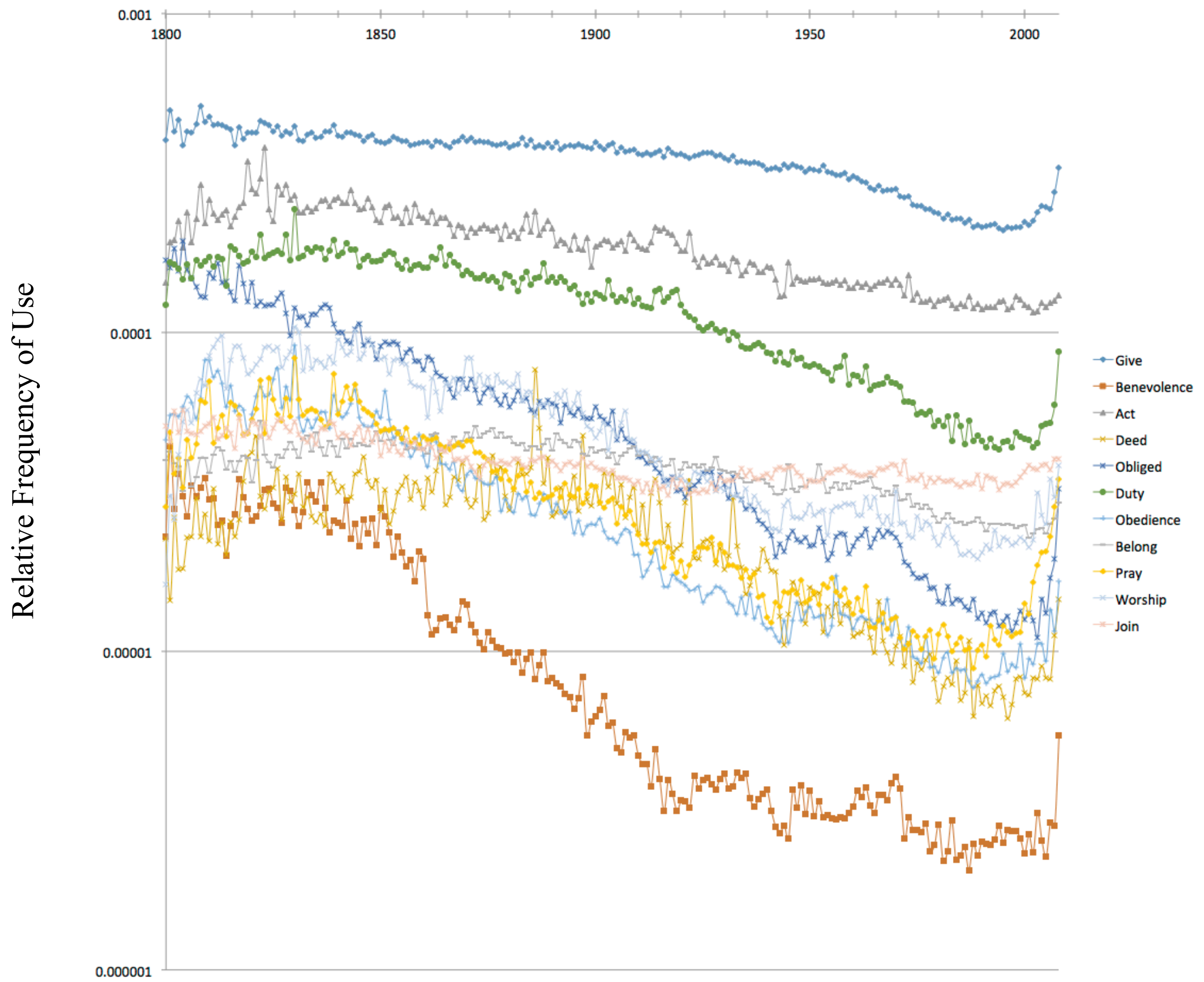

Figure 2, Individualistic indicators that trend away from individualistic values between 1980 and 2008

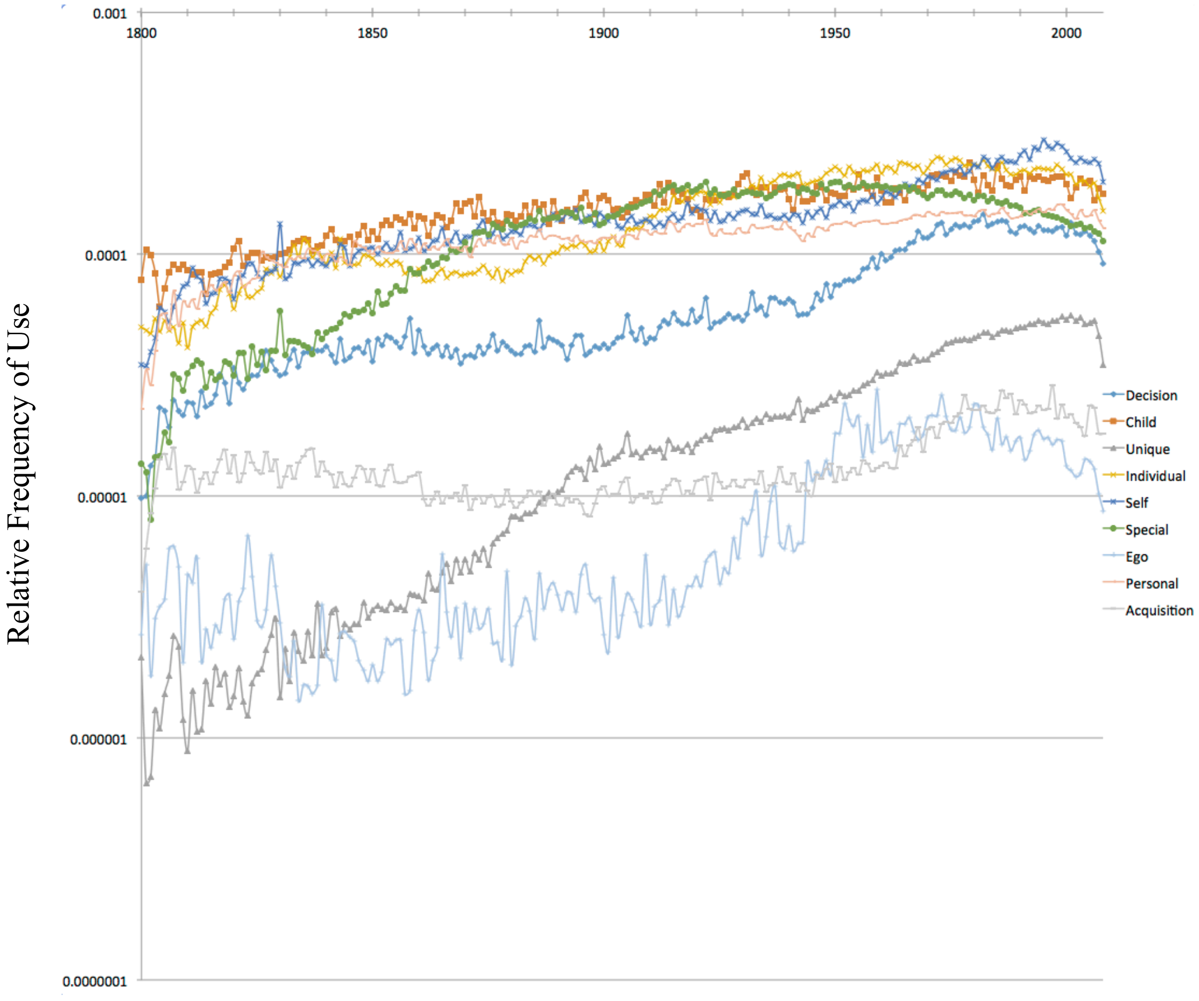

Figure 3, Collectivistic indicators that trend away from collectivistic values between 1980 and 2008

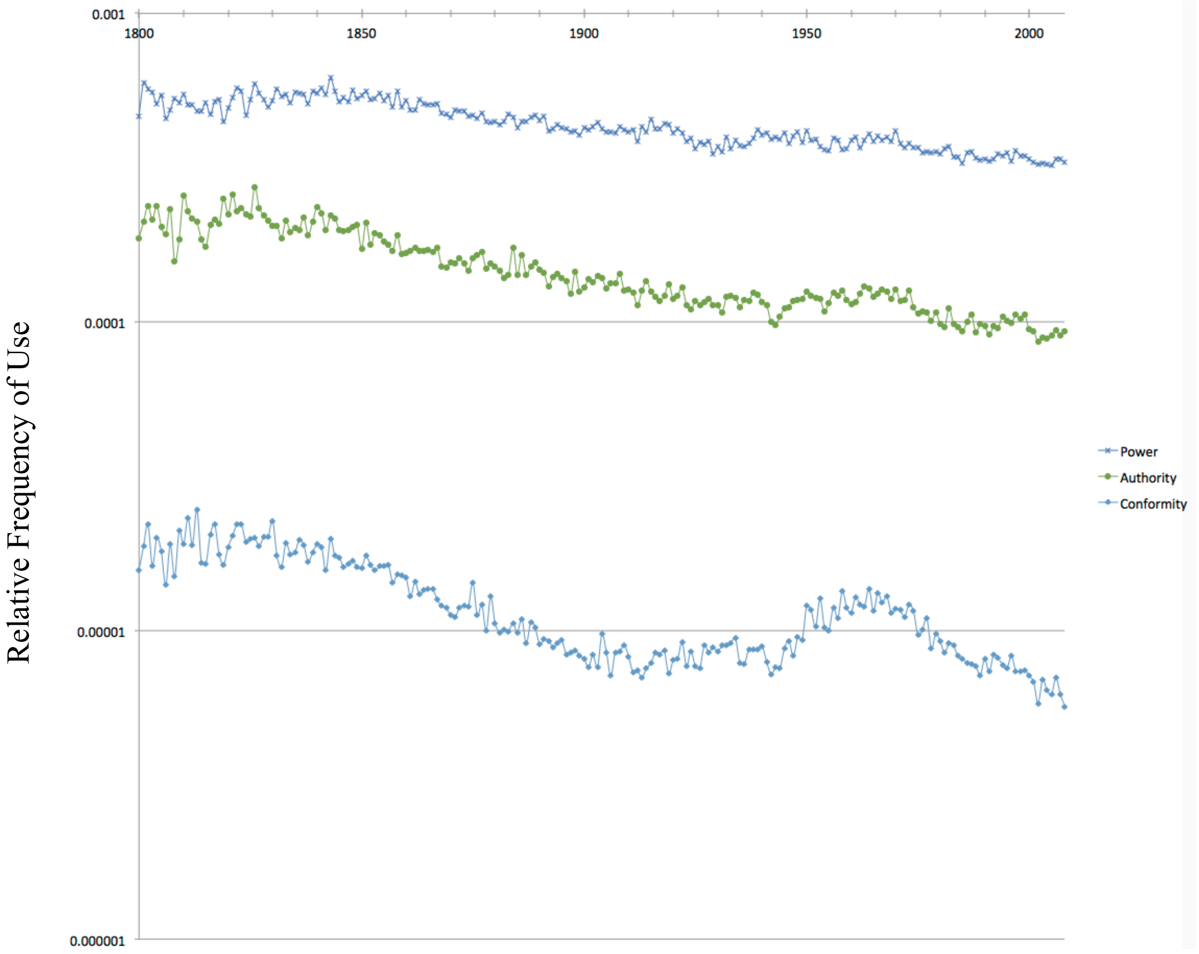

Figure 4, Individualistic indicators that trend toward individualistic values between 1980 and 2008

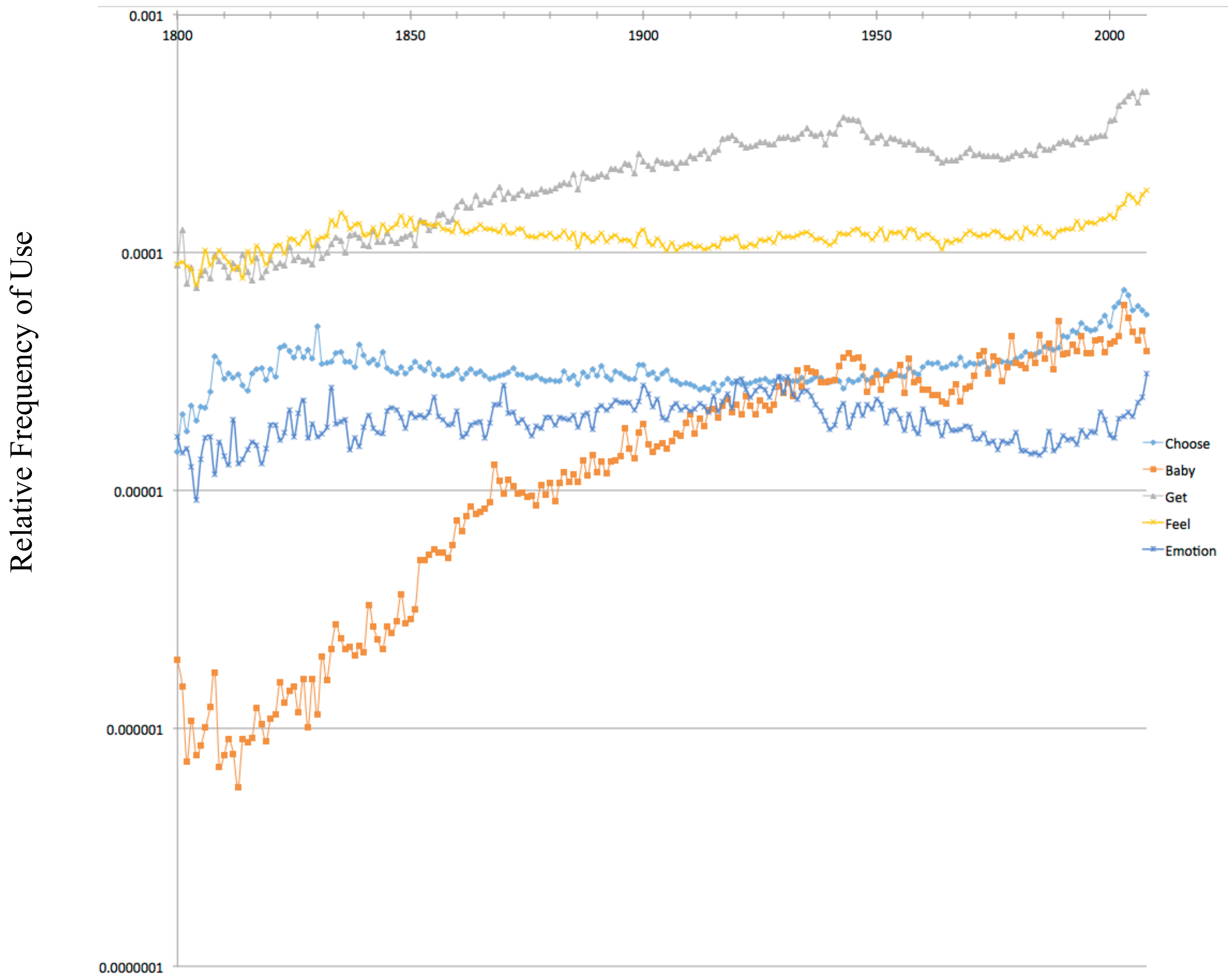

Supplement: Supplementary file 1 [file Appendix.pdf]
